# Complete Chloroplast Genome Sequence of Phagomixotrophic Green Alga *Cymbomonas tetramitiformis*

**DOI:** 10.1128/genomeA.00551-16

**Published:** 2016-06-16

**Authors:** Anchittha Satjarak, Amber E. Paasch, Linda E. Graham, Eunsoo Kim

**Affiliations:** a Department of Botany, University of Wisconsin-Madison, Madison, Wisconsin, USA; b Division of Invertebrate Zoology and Sackler Institute for Comparative Genomics, American Museum of Natural History, New York, New York, USA

## Abstract

We report here the complete chloroplast genome sequence of *Cymbomonas tetramitiformis* strain PLY262, which is a prasinophycean green alga that retains a phagomixotrophic mode of nutrition. The genome is 84,524 bp in length, with a G+C content of 37%, and contains 3 rRNAs, 26 tRNAs, and 76 protein-coding genes.

## GENOME ANNOUNCEMENT

*Cymbomonas tetramitiformis* is a marine prasinophycean green alga that bears a single chloroplast and lacks a cell wall (1). Within the prasinophytes, a paraphyletic assemblage of green algae that typically display features that are considered plesiomorphic for the *Chloroplastida* (green algae plus land plants), *C. tetramitiformis* falls into the order *Pyramimonadales* (prasinophyte clade I), which also includes *Halosphaera*, *Pyramimonas*, and *Pterosperma* (2). Unlike most other green algae, *C. tetramitiformis* retains the capacity to feed on bacteria while harvesting energy via photosynthesis (3).

*C. tetramitiformis* PLY262, acquired from the Plymouth Culture Collection of Marine Microalgae, was grown in f/2-Si at 18°C with a 12-h light/dark cycle. The total algal DNA was extracted using a Qiagen QIAamp minikit, according to the tissue-DNA protocol, and sent to the New York Genome Center (New York, NY) for whole-genome shotgun library preparation and sequencing on the Illumina MiSeq platform. A total of 19,183,050 paired sequences, each up to 300 bp in length, were generated. The raw reads were trimmed by Trimmomatic version 0.33 (4) in order to have a minimum quality score of 28 on the Phred 64 scale. The chloroplast genome was constructed by MIRA version 4.0.2 and MITObim version 1.8 (5) using the partial sequence of the *C. tetramitiformis* rbcL gene (GenBank accession no. L34687.1 [6]) as bait. The fold coverage of every position of the genome was calculated by mapping the trimmed reads to the newly assembled chloroplast genome using BWA nonmodel species alignment version 0.7.4 (7) and Bedtools genome coverage BAM version 2.19.1 (8) implemented in iPlant Collaborative (9). Open reading frames with length of at least 90 bp were predicted by conducting a BLAST search against the NCBI nonredundant protein database. tRNAs and rRNAs were predicted using tRNAscan-SE version 1.21 (10) and RNAmmer version 1.2 (11), respectively.

The complete chloroplast genome sequence was assembled into a circular-mapping molecule of 84,524 bp in length (1,200-fold coverage), with a G+C content of 37%. The genome was annotated with a total of 105 genes, including 76 protein-coding genes, 26 tRNAs, and 3 rRNAs. The region that contains three rRNAs and two tRNAs appears twice in the genome in the inverted orientation, the structure that is also found in the chloroplast DNA (cpDNA) of other prasinophyte and streptophyte algae (12). No introns were detected. A notable feature of *C. tetramitiformis*, unlike the closely related *Pyramimonas parkeae* (13), is the absence of all three genes (*chlL*, *chlB*, and *chlN*) encoding subunits of light-independent protochlorophyllide oxidoreductase (LIPOR) either in the chloroplast or nuclear genome (GenBank accession no. LGRX00000000). In fact, the loss of this gene set has been reported from cpDNA of a diverse range of photosynthetic eukaryotes, including the prasinophytes *Micromonas pusilla* and *Ostreococcus tauri*, as well as members of chlorarachniophytes, euglenids, rhodophytes, cryptophytes, haptophytes, stramenopiles, dinoflagellates, and chromerids (14). The oxygen sensitivity of LIPOR (15) may at least partially explain such multiple independent losses of LIPOR genes in response to the increased level of atmospheric oxygen since the origins of eukaryotic algae.

### Nucleotide sequence accession number.

The assembled chloroplast genome sequence of *C. tetramitiformis* has been archived at GenBank with the accession number KX013545.
